# Sixteenth Annual Convention

**Published:** 1865-07

**Authors:** 


### AMERICAN MEDICAL ASSOCIATION.

SIXTEENTH ANNUAL CONVENTION.

PROCEEDINGS OF TUESDAY, THE FIRST DAY.

The American Medical Association began its Sixteenth Annual Convention at the State House, Boston, in the Hall of the House of Representatives, on Tuesday, the 6th inst. A large number of delegates and members were present, occupying the entire floor of the Hall, while the galleries were occupied by spectators. The officers of the Convention were as follows:—

*President.*—N. S. Davis, M.D., of Illinois.

*Vice-Presidents.*—Wm. H. Mussey, M.D., of Ohio; Worthington Hooker, M.D., of Conn.; William Whelan, M.D., of District of Columbia; J. E. B. Heintze, M.D., of Maryland.

*Secretary.*—WILLIAM B. ATKINSON, M.D., of Philadelphia.

*Assist.-Secretary.*—HORATIA R. STORER, M.D., of Boston.

*Treasurer.*—CASPER WISTAR, M.D., of Philadelphia.

Considerable time was occupied in the registration of the names of delegates, under the direction of Dr. J. N. Borland, of the Committee of Arrangements. At about half-past 10 o'clock, the Convention was called to order, and prayer was offered by Rev. Dr. Lothrop, after which Dr. Henry J. Bigelow, of Boston, delivered the address of welcome.

